# Diet-Related Risk Factors for Incident Hypertension During an 11-Year Follow-Up: The Korean Genome Epidemiology Study

**DOI:** 10.3390/nu10081077

**Published:** 2018-08-13

**Authors:** Hye Ah Lee, Hyesook Park

**Affiliations:** 1 Clinical Trial Center, Mokdong Hospital, Ewha Womans University, 1071, Anyangcheon-ro, Yangcheon-ku, Seoul 07985, Korea; 2 Department of preventive medicine, College of Medicine, Ewha Womans University, 1071, Anyangcheon-ro, Yangcheon-ku, Seoul 07985, Korea

**Keywords:** dietary risk factors, hypertension, population-attributable fraction, prospective cohort study

## Abstract

Using long-term follow-up cohort data from the Korean Genome Epidemiology Study, we assessed the dietary risk factors for incident hypertension (HTN). In total, 6792 subjects (3300 males and 3492 females) aged 40–69 years were included in the study. Physician-diagnosed HTN self-reported by the participants was used as the outcome. Daily intake of 20 food groups was assessed while using a dish-based semi-quantitative food-frequency questionnaire. After controlling for known risk factors, the food groups that were most closely associated with HTN were identified by forward stepwise selection while using the Cox proportional hazards model. The median follow-up period was 11.5 years (interquartile range, 6.0–11.7 years) and the incidence of HTN was 20 per 1000 person-years. Older age, obesity, lower education level, high alcohol intake, and having at least one parent with HTN were associated with the risk for HTN. In addition, a high intake of salted seafood and a low intake of eggs and meat were independently associated with the incidence of HTN after controlling for the known risk factors. Those in the top quartile of salted seafood intake had a 28% greater risk for incident HTN than those in the bottom quartile. The population-attributable fraction of three dietary factors accounted for 29.0% of the incidence of HTN. A high intake of salted seafood and a low intake of eggs and meat were associated with a greater risk for HTN.

## 1. Introduction

Hypertension (HTN) is a major concern in public health, as it is a risk factor for cardiovascular disease (CVD) with a high prevalence [1]. The World Health Organization (WHO) reported that high blood pressure kills nine million people annually [1]. According to the Korea National Health and Nutrition Examination Survey (KNHANES), about 30% of the Korean population aged ≥30 years reported having HTN [2], which is similar to the rates in other high-income countries [1,3].

Prevention of HTN would be most effective in reducing the burden of both HTN and CVD. Thus, it is necessary to explore the risk factors for HTN, in terms of primary prevention. Accumulated evidence derived from a large-scale cohort study suggested several risk factors for HTN, including parental HTN [4,5], smoking [4], and a high body mass index (BMI) [4]; however, dietary factors were not considered. Diet-related factors play an important role in the prevention and management of HTN, and they are potentially modifiable. The Dietary Approaches to Stop Hypertension (DASH) diet, which includes a high proportion of fruits, vegetables, and low-fat dairy products, and a low proportion of total and saturated fats and sugar, is reportedly effective for controlling blood pressure in patients with HTN [6] and preventing CVD [7]. Furthermore, evidence implies that specific food groups (e.g., fruits and vegetables [8,9,10]) and nutrients (e.g., saturated fat and sodium [8,11]) are associated with HTN risk.

Identification of dietary factors that are associated with HTN is hampered by interactions among foods and nutrients. Therefore, most studies have focused on specific food groups or nutrients. Although identifying nutrients that are related to disease aids in an understanding of the underlying biological pathways, making recommendations to the general population regarding healthy-eating habits is problematic.

This study used the long-term follow-up cohort data of the Korean Genome Epidemiology Study (KoGES). We sought dietary risk factors related to incident HTN, taking into account the known risk factors. We also estimated the population-attributable fraction (PAF) of dietary factors for the incidence of HTN. 

## 2. Materials and Methods

### 2.1. Study Subjects

This study was conducted using community-based cohort data of the KoGES. The KoGES consists of six prospective cohort studies in two categories: population-based and gene-environment model studies [12]. The community-based cohort in the population-based study that began in 2001–2002 involved persons (*n* = 10,030) aged 40–69 years who resided in communities in Ansung (*n* = 5018; rural region) and Ansan (*n* = 5012; industrial region) in Gyeonggi Province. All of the participants initially provided informed consent. This study was of a prospective cohort design and sought risk factors for non-communicable diseases, including HTN and diabetes, in Koreans. A follow-up survey has been conducted every two years by trained technicians/interviewers, involving questionnaires, anthropometric measurements, and serological/urine tests. Currently, data are available for all surveys up to the seventh (2013–2014). We accessed the data with approval from the National Research Institute of Health. The follow-up rate of the seventh survey (6th follow-up survey) was 62.8% [12]. Comparisons of the baseline characteristics of the follow-up participants and non-participants in the KoGES community-based cohort have already been described elsewhere [12]. Detailed information about the cohort is available online [13]. 

The following subjects were excluded from this study: those with missing dietary data (*n* = 326), with a daily calorie intake <500 kcal or >5000 kcal (*n* = 84), and/or with a history of any type of cancer, myocardial infarction, stroke, coronary artery disease, congestive heart failure, lipidemia, or HTN in the baseline survey (*n* = 2828). The subjects participated in at least one follow-up survey. Thus, this study comprised 6792 persons (males, 3300; females, 3492). Those that were excluded from this study were slightly older, had a slightly lower average socioeconomic status, and were less healthy (e.g., higher low-density lipoprotein cholesterol (LDL-c) levels, higher proportion of BMI ≥ 23.0 kg/m^2^, etc.) than those that were included in this study (data not shown). The study protocol was approved by the Institutional Review Board of Ewha Womans University Hospital. 

### 2.2. Assessment of Hypertension

Subjects were followed from 2001–2002 to their sixth follow-up visit (2013–2014). HTN was identified when patients reported that this condition had been diagnosed by a physician. The follow-up period covered the age at entrance into the study to the age at which HTN was diagnosed or the age at the last follow-up if hypertension did not develop.

### 2.3. Dietary Data

At baseline, a dietary survey was conducted by a trained interviewer while using a food frequency questionnaire (FFQ) consisting of 103 food items. The validity and reliability of the FFQ are acceptable [14]. The semi-quantitative FFQ provided information on average dietary consumption for the past year using a nine-point scale (less than once per month or never, once per month, 2- to 3-times per month, 1- to 2-times per week, 3- to 4-times per week, 5- to 6-times per week, once per day, twice per day, and three-times per day) and three levels to represent the amount that is consumed with reference to a standard amount (less, standard, and more). Of the 103 food items, similar items were categorized into 20 food groups; rice, noodles, bread, sugar, oil and fat, potatoes, soybean, nuts and seeds, Kimchi, vegetables, mushrooms, fruit, meat, eggs, fish, shellfish, salted seafood, seaweeds, dairy products, and drinks (Supplemental Table S1). The average daily consumption of each food group was estimated as the energy-adjusted intake (g/day) using the residual method. The energy-adjusted intake of each food group was divided into quintiles and analyzed. The cut-off values for quintiles of the intake of each food group are presented in Supplemental Table S2. 

### 2.4. Potential Risk Factors

Based on the evidence, we evaluated BMI [4], smoking status (non-smoker, ex-smoker, current smoker) [4,10], renal function [15], LDL-c [16], parental HTN (yes/no) [4,5], and diabetes at baseline (yes/no) [17] as potential risk factors. BMI was classified into two groups (<23 and ≥23 kg/m^2^) and reduced renal function was defined as an estimated glomerular filtration rate (eGFR) < 60 mL/min per 1.73 m^2^ [15]. The LDL-c level was estimated using the Friedewald formula: Total cholesterol (mg/dL) − high-density lipoprotein cholesterol (HDL-c, mg/dL) − (triglycerides (mg/dL)/5). Alcohol intake was also evaluated [17] and categorized, as described previously (non-intake or <15, 15–24.9, or ≥25 g/day) [18,19]. More than 15 g/day in women and ≥25 g/day in men can be considered as more than a moderate intake of alcohol. As socioeconomic factors, we evaluated the education level (graduated from middle school or lower, graduated from high school, and college or higher) and monthly income. However, these factors were closely correlated. Therefore, to avoid over-adjustment, only education level was considered in the analysis.

### 2.5. Statistical Analysis

Summary statistics are presented as means with standard deviations for normally distributed numerical data and as medians with interquartile ranges (IQRs) for numerical data with a skewed distribution. We assessed the normality of the distribution of variables with the Shapiro–Wilk test with a normal quantile-quantile plot. Categorical data are presented as numbers and percentages of subjects. To compare participants’ basic characteristics in terms of the HTN incidence, we used the Student’s *t*-test for normally distributed numerical data and the Chi-square test for categorical data. 

The incidence rate of HTN was estimated in units of 1000 person-years. The risk factors for HTN were assessed while using the Cox proportional hazards model. The effects of risk factors on the development of HTN were expressed as hazard ratios (HRs) with 95% confidence intervals (95% CIs). In terms of dietary risk factors, we treated intake quintiles for each food group as categorical variables. We compared the effects of dietary risk factors on HTN, according to intake using the low-intake group (Q1) as the reference. Through a crude model, we assessed the incidence risk of HTN by comparing high and low intake groups (Q5 vs. Q1). The food groups that satisfied *p* < 0.1 were included in the forward stepwise selection along with the potential risk factors. As potential risk factors, the effects of BMI status, smoking status, alcohol drinking, renal function, low-density lipoprotein cholesterol, parental HTN, diabetes, education level, sex, age, and survey region on the incidence of HTN were assessed simultaneously. Furthermore, to consider the correlation between foods, those foods that correlated significantly with the selected food groups were included in the model. We then further analyzed the potential independent effects of the selected foods. The assumptions of the statistical model were examined while using the Schoenfeld residuals method and satisfied. Next, multicollinearity among risk factors was assessed based on the variance inflation factor, and had a value <5.0. 

The PAFs of the risk factors were calculated as: (prevalence × (HR-1)) ÷ (prevalence × (HR-1) + 1) × 100. For the calculation of PAF, we used the top quintile of food intake as a reference group when there was a negative relationship between quintile of food intake and HTN. All of the statistical analyses were conducted using SAS ver. 9.4 software (SAS Institute, Cary, NC, USA) and R ver. 3.3.3 for Windows (R Foundation for Statistical Computing, Vienna, Austria). A two-sided *p*-value < 0.05 was considered indicative of statistical significance.

## 3. Results

The median follow-up period was 11.5 years (IQR, 6.0–11.7 years). The incidence rate of HTN among Koreans aged ≥40 years was 20 per 1000 person-years (cumulative incidence = 18 per 100 persons). Of the subjects, 48.6% were males and 48.2% resided in a rural region. More than half of the subjects had a lower educational level and one-third had a lower income level. At least one of the parents of about 14.3% of the subjects had HTN. More than one quarter of the subjects were current smokers, and 66.0% were overweight or obese. Subjects with HTN were slightly older and had a slightly lower average socioeconomic status. Moreover, 14.3% of those with HTN consumed ≥25 g/day of alcohol, which was higher than the proportion of those without HTN who did so (11.9%); this association reached borderline significance (*p* < 0.06). The distribution of participants according to smoking status did not significantly differ by HTN incidence (*p* = 0.41). More than three quarters of subjects with HTN were obese at the baseline. The percentage of participants with one or more parent with HTN was higher in subjects with HTN than in those without HTN (18.4% vs. 13.4%, *p* < 0.0001) (Table 1).

**Figure 1:**
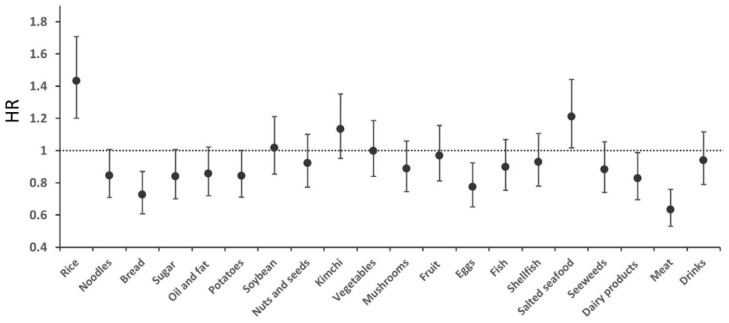
Hazard ratios of the 20 food groups for incident hypertension (HTN). Circles and bars indicate hazard ratios with 95% confidence intervals. The hazard ratios (with 95% confidence intervals) for incident hypertension (HTN) are shown for comparisons of the top and bottom quintile (Q5 vs. Q1). Individual food group intakes were estimated after controlling for total energy using the residual method.

The average daily intake of the 20 food groups is shown in Supplemental Table S3. The mean daily total energy intake was 1952.8 kcal (±620.0). The intakes of dairy products and eggs were slightly higher in the subjects without than in those with incident HTN, and vice versa for the intake of salted seafood. Figure 1 shows the HRs and 95% CIs of the comparison between the top and bottom quintiles (Q5 vs. Q1) of the 20 food groups with respect to the development of HTN (crude model). Of the 20 food groups, a high intake of rice and salted seafood was associated with an increased risk of HTN, whereas a high intake of meat, eggs, and dairy products was inversely associated with the incidence of HTN (Figure 1). 

Subjects who were older, obese, had a lower educational level, high alcohol intake, and at least one parent with HTN had a significantly greater risk for developing HTN. Current smokers, reduced renal function, and diabetes had a HR > 1.0 for the risk of incident HTN, but this did not reach statistical significance. Regarding dietary factors, stepwise selection led to the inclusion of salted seafood, eggs, and meat in the known and dietary risk factors model (Table 2). Subjects with a BMI ≥ 23.0 kg/m^2^ had a 1.71-fold higher risk for HTN. Having a parent with HTN was also associated with the risk for incident HTN (HR, 1.52; 95% CI, 1.31–1.77). A high intake of alcohol was also associated with a significant risk for HTN. When comparing the highest and lowest intake groups (Q5 vs. Q1), high salted seafood intake was associated with the development of HTN, evidenced by an HR of 1.28 (95% CI, 1.07–1.55), after controlling for the known risk factors with eggs and meat intake. However, high eggs and meat intake was associated with a reduced risk for incident HTN (Table 2). In addition, the effects remained, even after adjusting for correlated food groups (data not shown). 

In terms of PAFs, 31.8% of incident HTN was attributable to overweight or obesity, followed by parental hypertension (7.0%) and high alcohol consumption (3.8%). Regarding dietary factors, low meat intake (18.6%), low egg intake (5.0%), and high salted seafood intake (5.4%) contributed to incident HTN. Therefore, 42.6% and 29.0% of incident HTN was attributable to known and dietary risk factors, respectively (Table 3).

## 4. Discussion

Using long-term follow-up data of middle-aged Koreans, our results revealed that meat, eggs, and salted seafood intake were independently associated with the incidence of HTN after controlling for known risk factors. Moreover, 29.0% of incident HTN was attributable to a low intake of meat and eggs and a high intake of salted seafood. 

According to a global burden of disease study, dietary factors and high systolic blood pressure were ranked second and third among factors contributing to the 2016 global burden of disease, accounting for 9.6% and 8.9% of the total disability-adjusted life years (DALY), respectively. High systolic blood pressure was also the most significant contributor to incident CVD and mortality [20]. In the 2012 Korean Burden of Disease Study, the DALYs of cardiovascular and circulatory diseases ranked second among non-communicable diseases [21]. Therefore, it is important to maintain a normal blood pressure. Prevention or non-pharmacological treatment of HTN is managed by lifestyle modification, such as losing weight, increasing physical activity, and a healthy diet. 

Regarding a healthy diet, much evidence suggests that reducing salt intake is beneficial to health, and the WHO recommends ingesting less than 2000 mg sodium per day [1]. Although a “less salt” campaign is underway in Korea [22], around 80% of the population consumes more than the recommended standard based on the 2016 KNHANES [23]. Excessive salt intake is also a public health issue in other countries [24]. The mechanism underlying the role of sodium in the development of HTN is complex, but epidemiological or experimental studies have shown that blood pressure can be reduced by limiting sodium intake [11]. In this study, a high intake of salted seafood was associated with a 28% higher risk for incident HTN. Consistent with our study, dietary patterns of alcohol consumption and a high intake of salted fermented seafood were significantly associated with the prevalence of pre-hypertension and hypertension in a cross-sectional study in Korea [25]. Salted seafood is popular in Korea and it has a high sodium content [26]. Salted seafood intake may differ according to age, region, and individual preference [27]. Seasonings, such as soy sauce, also have a high salt content and are commonly used in Asia [28]. Typical sodium-content of foods consumed varies among countries due to differences in food preparation, cooking methods, and food preferences [28]. Therefore, with an in-depth survey of dietary sources of sodium, it would be necessary to develop an intervention strategy that is tailored to the characteristics of different countries.

As regards unfavorable effects that are associated with low levels of consumption, eggs and meat appeared significant. Although eggs have a high cholesterol content, they also contain nutrients that protect against heart disease. These include carotenoids (lutein and zeaxanthin), which have antioxidant and anti-inflammatory activities, and arginine, which improves cardiovascular health by dilating blood vessels [29]. In parallel with our results, a recent prospective study involving Iranian adults reported an inverse association between egg intake and the incidence of hypertension (top vs. bottom tertiles, OR, 0.54; 95% CI, 0.32–0.91) [9,30]. In a clinical trial in metabolic syndrome patients, the supplementation of egg-derived phospholipids improved endothelial function and decreased systolic blood pressure [31]. A systematic review also reported that egg intake did not have a negative effect on CVD, except in subjects with diabetes [29]. Moreover, cholesterol derived from eggs did not have a deleterious effect on the blood lipids profile of the general population, and so a general restriction of egg consumption is not recommended [32].

Regarding meat consumption, a previous study using the same data reported that the risk for CVD decreases with increasing the intake of unprocessed meat, particularly poultry [33]. The effects of consumption of unprocessed red meat on the risk for CVD differ between Asia and the United States (US) and Europe [34,35]. This can be explained by differences in the actual intake, which was lower than in Western countries [36]. Red meat is a source of protein, as well as numerous vitamins and minerals [37]. Although the Spearman correlation coefficients were weak, meat intake was positively correlated with intakes of fish and shellfish (data not shown). Accordingly, lack of nutrients due to insufficient meat intake would have had a negative association with the development of HTN in this study. However, according to the United Nations Food and Agriculture Organization, the prevalence of animal-based diets is increasing in Korea [38], particularly in young adults [39]. Changes in meat intake may influence the association with future health risks that differ from these results. 

There is evidence that smoking causes cardiovascular damage, but our study did not shown an association. Other studies have also reported null or inverse [5] associations. Even after further analysis, excluding smokers, the impact of meat, eggs, and salted seafood was more pronounced and they did not differ significantly from the given results (data not shown). Moreover, the survey region was independently associated with incident HTN in this study; this seems to have been caused by differences in access to healthcare and foodstuffs, which may have led to differences in the HTN status of rural and urban residents.

In this study, we treated self-reported physician-diagnosed HTN as an outcome. Although questions about the validity of self-reported HTN have been raised, there is still a misclassification problem due to measurement errors when the measurements are simply applied. A recent study using KNHANES data found that the accuracy of self-reported HTN in Koreans over 50 years of age was relatively high [40]. As a sub-analysis, we assessed the final model by applying clinical criteria (SBP ≥ 140 mmHg or DBP ≥ 90 mmHg) and drug treatment as the definitions of incident HTN, and the influence of meat and eggs remained significant, even though incident HTN was defined in terms of various combinations (data not shown). 

Although our results are derived from a long-term cohort study, several points should be considered in their interpretation. First, the community-based cohort was not representative of the Korean population, leading to a lack of generalizability of the results. In addition, as survey instruments and food groupings were defined according to subject characteristics, they might not be comparable with populations in other countries. Second, uncertainty resulting from measurement errors and self-reported disease information might have influenced the results. Third, the misclassification bias caused by this uncertainty might have resulted in attenuated effects, but our results are supported by existing evidence. It is also necessary to consider the possibility of bias due to loss to follow-up. Residual confounding factors due to unmeasured risk or other dietary information, such as serum uric acid levels, use of statins, and eating time and duration, may have influenced the results. Changes in food intake over time can affect the association and act as a major confounder, but they were not considered. Finally, there remains the issue of multiple comparisons, which is related to the possibility of false positives. Thus, there is a need for additional studies to assess the validity of our results.

Nevertheless, this study also had several strengths, being a large-scale, long-term observational study that was conducted in Asia, where there has been a lack of research thus far. Our results were obtained using the average dietary intake data extracted from a validated instrument. In view of the possibility of the use of dietary control as a therapeutic strategy, we excluded subjects with cancer, CVD, and lipidemia (including hypertension) at baseline. Accordingly, relatively healthy people remain in the analysis, and the health status effects may have been undervalued. We also identified food groups that are associated with the incidence of HTN after simultaneously controlling for dietary and other known risk factors. The PAF results imply that weight status was most closely associated with the incidence of HTN, followed by meat intake. However, in considering action plans for preventing HTN, focusing on a healthy lifestyle and diet is probably more important than any one risk factor.

## 5. Conclusions

In conclusion, maintaining a healthy lifestyle and a balanced diet would reduce the overall disease burden. Using long-term follow-up data, we showed that a high intake of salted seafood and low intake of eggs and meat were associated with an increased incidence of HTN, even after adjusting for known risk factors. Our study focused on the foods related to incident HTN; thus, research on dietary patterns is still necessary. Furthermore, further studies are needed to confirm our results and determine the appropriate intakes of various types of foods.

## Figures and Tables

**Table 1:** Characteristics of the study subjects.

	Total (*n* = 6792)	Without HTN Incidence (*n* = 5580)	With HTN Incidence (*n* = 1212)	*p* Value
**Age, years**	51.2 ± 8.7	50.8 ± 8.7	53.2 ± 8.5	<0.0001 ^a^
**Male, %**	3300 (48.6)	2712 (48.6)	588 (48.5)	0.98 ^b^
**Rural area, %**	3275 (48.2)	2676 (48.0)	599 (49.4)	0.37 ^b^
**Education level, %**				
Less than middle school graduate	3665 (54.2)	2951 (53.1)	714 (59.2)	<0.001 ^b^
Graduated high school	2180 (32.3)	1820 (32.8)	360 (29.8)	
Some college or higher	915 (13.5)	782 (14.1)	133 (11.0)	
**Monthly income (KRW), %**				
<1,000,000	2177 (32.5)	1727 (31.4)	450 (37.7)	<0.001 ^b^
1,000,000–1,999,999	1996 (29.8)	1658 (30.1)	338 (28.3)	
≥2,000,000	2522 (37.7)	2116 (38.5)	406 (34.0)	
**BMI status, %**				
BMI < 23.0 kg/m^2^	2307 (34.0)	2019 (36.2)	288 (23.8)	<0.0001 ^b^
BMI ≥ 23.0 kg/m^2^	4484 (66.0)	3560 (63.8)	924 (76.2)	
**Smoking status, %**				
Never smokers	3918 (58.2)	3216 (58.1)	702 (58.5)	0.41 ^b^
Former smokers	1022 (15.2)	828 (15.0)	194 (16.1)	
Current smokers	1793 (26.6)	1488 (26.9)	305 (25.4)	
**Alcohol intake, %**				
Non-drinking	3431 (51.8)	2817 (51.8)	614 (51.7)	0.06 ^b^
<15 g/day	1929 (29.1)	1610 (29.6)	319 (26.9)	
15–24 g/day	451 (6.8)	366 (6.7)	85 (7.1)	
≥25 g/day	816 (12.3)	646 (11.9)	170 (14.3)	
**Renal function, %**				
<eGFR 60 mL/min per 1.73 m^2^	199 (2.9)	148 (2.7)	51 (4.2)	<0.01 ^b^
**LDL-c (mg/dL)**	113.1 ± 32.5	112.7 ± 32.5	114.9 ± 32.4	0.03 ^a^
**Parental HTN, %**				
Yes	971 (14.3)	748 (13.4)	223 (18.4)	<0.0001 ^b^
**Diabetes, %**				
Yes	313 (4.6)	237 (4.3)	76 (6.3)	<0.01 ^b^

SD, standard deviation; HTN, hypertension; BMI, body mass index; KRW, Korean Won; eGFR, estimated glomerular filtration rate; LDL-c, low-density lipoprotein cholesterol. ^a^ The *p* value was derived using the Student’s *t*-test for the comparison of the mean difference between two groups. ^b^ The *p* value was derived using the Chi-square test to compare the distributions among groups.

**Table 2:** Effects of potential risk factors on the incidence of HTN ^†^.

	Known Risk Factors			Known Risk Factors + Dietary Risk Factors (High Group vs. Low Group)		
	HR	95% CI		HR	95% CI	
**Age**	1.04	1.03	1.05	1.04	1.03	1.05
**Sex** (ref. Female)	1.00	0.83	1.22	1.03	0.84	1.25
**Rural area**	0.85	0.74	0.97	0.84	0.73	0.96
**Education level** (ref. some college or higher)						
Graduated middle school	1.30	1.06	1.61	1.29	1.05	1.59
Graduated high school	1.17	0.95	1.43	1.18	0.96	1.44
**BMI status** (ref. < 23 kg/m^2^)						
BMI ≥ 23 kg/m^2^	1.72	1.50	1.97	1.71	1.49	1.96
**Smoking status** (ref. Never smokers)						
Former smokers	1.04	0.83	1.29	1.04	0.84	1.30
Current smokers	1.09	0.89	1.33	1.10	0.90	1.34
**Alcohol intake** (ref, non-drinker)						
<15 g/day	0.98	0.85	1.13	0.99	0.86	1.15
15–24	1.06	0.83	1.36	1.09	0.85	1.40
≥25 g/day	1.31	1.07	1.60	1.32	1.08	1.62
**Renal function** (eGFR ≥ 60 mL/min per 1.73 m^2^)						
eGFR < 60	1.29	0.97	1.71	1.30	0.98	1.73
**LDL-c**	1.00	1.00	1.00	1.00	1.00	1.00
**Parental HTN** (ref. no)	1.52	1.30	1.76	1.52	1.31	1.77
**Diabetes** (ref. no)	1.24	0.98	1.57	1.24	0.97	1.57
**Dietary factors** ^‡^						
Meat intake				0.68	0.56	0.83
Eggs intake				0.79	0.66	0.95
Salted seafood intake				1.28	1.07	1.55

BMI, body mass index; eGFR, estimated glomerular filtration rate; LDL-c, low-density lipoprotein cholesterol; HR, hazard ratio; 95% CI, 95% confidence interval. ^†^ Hazard ratios with 95% confidence intervals were estimated by taking into account the variables presented in the table simultaneously in each statistical model. ^‡^ Quintiles of food group intake were applied as categorical variables in the analysis.

**Table 3:** Population-attributable risks of potential risk factors for incident HTN.

	Subgroup	Exposure%	HR	95% CI		PAF (%)	∑PAF (%)
**Known risk factors**							
**BMI status**	BMI ≥ 23 kg/m^2^	66.0	1.71	1.49	1.96	31.8	42.6
**Alcohol intake**	≥25 g/day	12.3	1.32	1.08	1.62	3.8	-
**Parental HTN**	Yes	14.3	1.52	1.31	1.77	7.0	-
**Dietary risk factors ^†^**							
**Meat (g/day)**	<26.3	20.0	1.46	1.20	1.78	8.5	29.0
	26.3–<41.7	20.0	1.28	1.05	1.56	5.4	-
	41.7–<58.3	20.0	1.25	1.03	1.52	4.7	-
	58.3–<83.7	20.0	1.19	0.98	1.44	-	-
	≥83.7	20.0	1.00	-	-	-	-
**Eggs (g/day)**	<2.6	20.0	1.27	1.05	1.52	5.0	-
	2.6–<6.2	20.0	1.00	0.82	1.21	-	-
	6.2–<11.0	20.0	1.13	0.94	1.36	-	-
	11.0–<21.3	20.0	1.02	0.84	1.23	-	-
	≥21.3	20.0	1.00	-	-	-	-
**Salted seafood (g/day)**	0	20.0	1.00	-	-	-	-
	0<–<0.5	20.0	1.05	0.87	1.27	-	-
	0.5–<1.0	20.0	1.11	0.91	1.36	-	-
	1.0–<2.6	20.0	1.12	0.92	1.37	-	-
	≥2.6	20.0	1.28	1.07	1.55	5.4	-

BMI, body mass index; eGFR, estimated glomerular filtration rate; HTN, hypertension; HR, hazard ratio; 95% CI, 95% confidence interval; PAF, population-attributable fraction. Hazard ratios with 95% confidence intervals were estimated by the Cox proportional hazards model, which included age, sex, rural region, educational level, obesity, smoking status, alcohol intake, renal function, low-density lipoprotein cholesterol, parental HTN, and diabetes, as well as salted seafood, eggs, and meat intake. ^†^ The average daily consumption was estimated after controlling for total energy using the residual method.

## Supplementary Materials

The following are available online at http://www.mdpi.com/2072-6643/10/8/1077/s1, Table S1: Composition of the 20 food groups; Table S2: Criteria for subgrouping of daily intake (g/day) of the 20 food groups; Table S3: Average daily intakes (g) of the 20 food groups.
