# Supplementary material for: Diet-Related Risk Factors for Incident Hypertension During an 11-Year Follow-Up: The Korean Genome Epidemiology Study
Source: Nutrients. 2018 Aug 13;10(8):1077. doi: 10.3390/nu10081077 (PMC6115751; doi:10.3390/nu10081077)
Supplement: Supplementary file 1 [file nutrients-10-01077-s001.pdf]

Supplemental table S1. Composition of the 20 food groups

Food group	Food items
Rice	Cooked well-milled rice, cooked rice with barley, cooked rice with other cereals, parched cereal powder, rice cakes (plain rod shape), other rice cakes, cereals
Noodles	Ramen, noodles with soup, chajangmyon, buckwheat vermicelli/buckwheat noodle, dumpling
Bread	Loaf bread, bread with small red beans, other breads, pizza/fast food, cakes
Sugar	Snacks, candy/chocolate, coffee sugar, coffee cream
Oil and fat	Butter/margarine
Potatoes	Potatoes, sweet potatoes, starch vermicelli, starch jelly
Soybean	Soybean, tofu, stew with soybean pastes, soybean milk
Nuts and seeds	Nuts
Kimchi	Kimchi, Korean cabbage, kakduki/small radish kimchi, kimchi with liquid, other kimchi (green onion/kodulbbagi/mustard leaves), pickles
Vegetables	Green pepper, red pepper leaves, spinach, lettuce, perilla leaf, leek/water dropwort, green-yellow vegetables, radish/salted radish, doraji/deoduck (kinds of white root), onion, cabbages, cucumber, bean sprouts, carrot, pumpkin gruel/pumpkin juice, young pumpkin, vegetable juice, bracken/sweet potato stalk, tomato/tomato juice
Mushrooms	Oyster mushroom, other mushrooms
Fruit	Persimmon, dried persimmon, tangerine, muskmelon/melon, banana, pear, apple/apple juice, orange/orange juice, watermelon, peach/plum, strawberry, grape/grape juice
Meat	Pan-roast pork, pork belly, braised pork, pan-roast beef ribs, thick beef soup/hard-boiled beef ribs, chicken/chicken leg/chicken wing, ham/sausage, dog meat, edible viscera
Eggs	Eggs
Fish	Sushi, hair tail, eel, yellow croaker, Alaska pollack, mackerel/Pacific saury/Spanish mackerel, dried anchovy
Shellfish	Cuttlefish/octopus, tuna, canned, fish paste/crab flavored, crab, clam (small ark shell/little neck clam/clam meat), oyster, shrimp
Salted seafood	Salt-fermented seafood (fish, shrimp, etc.)
Seaweeds	Laver, kelp/sea mustard
Dairy products	Milk, yoghurt, ice cream, cheese
Drinks	Carbonated drinks, coffee, green tea, sikhae, citron tea

Supplemental table S2. Criteria for subgrouping of daily intake (g/day) of the 20 food groups†

Food group	Q1	Q2	Q3	Q4	Q5
Rice	< 547.7	547.7 ≤ -< 647.9	647.9 ≤ -< 712.7	712.7 ≤ -< 778.2	≥ 778.2
Noodles	< 29.8	29.8 ≤ -< 51.7	51.7 ≤ -< 75.9	75.9 ≤ -< 112.8	≥ 112.8
Bread	< 1.8	1.8 ≤ -< 7.4	7.4 ≤ -< 12.4	12.4 ≤ -< 23.4	≥ 23.4
Sugar	< 3.6	3.6 ≤ -< 7.7	7.7 ≤ -< 13.3	13.3 ≤ -< 23.2	≥ 23.2
Oil and fat	0	0 < -< 0.003	0.003 ≤ -< 0.02	0.02 ≤ -< 0.04	≥ 0.04
Potatoes	< 6.8	6.8 ≤ -< 13.0	13.0 ≤ -< 20.2	20.2 ≤ -< 33.7	≥ 33.7
Soybean	< 16.2	16.2 ≤ -< 26.1	26.1 ≤ -< 37.7	37.7 ≤ -< 58.6	≥ 58.6
Nuts and seeds	0	0 < -< 0.2	0.2 ≤ -< 0.4	0.4 ≤ -< 0.8	≥ 0.8
Kimchi	< 98.3	98.3 ≤ -< 152.1	152.1 ≤ -< 199.3	199.3 ≤ -< 289.7	≥ 289.7
Vegetables	< 65.8	65.8 ≤ -< 93.5	93.5 ≤ -< 122.8	122.8 ≤ -< 172.3	≥ 172.3
Mushrooms	< 1.4	1.4 ≤ -< 3.3	3.3 ≤ -< 6.3	6.3 ≤ -< 12.9	≥ 12.9
Fruit	< 97.1	97.1 ≤ -< 172.2	172.2 ≤ -< 247.8	247.8 ≤ -< 383.1	≥ 383.1
Meat	< 26.3	26.3 ≤ -< 41.7	41.7 ≤ -< 58.3	58.3 ≤ -< 83.7	≥ 83.7
Eggs	< 2.6	2.6 ≤ -< 6.2	6.2 ≤ -< 11.0	11.0 ≤ -< 21.3	≥ 21.3
Fish	< 11.4	11.4 ≤ -< 18.4	18.4 ≤ -< 26.1	26.1 ≤ -< 40.5	≥ 40.5
Shellfish	< 3.7	3.7 ≤ -< 7.3	7.3 ≤ -< 11.6	11.6 ≤ -< 18.8	≥ 18.8
Salted seafood	0	0 < -< 0.5	0.5 ≤ -< 1.0	1.0 ≤ -< 2.6	≥ 2.6
Seaweeds	< 0.7	0.7 ≤ -< 1.2	1.2 ≤ -< 1.8	1.8 ≤ -< 2.8	≥ 2.8
Dairy products	< 21.9	21.9 ≤ -< 51.5	51.5 ≤ -< 103.4	103.4 ≤ -< 202.8	≥ 202.8
Drinks	< 17.8	17.8 ≤ -< 36.4	36.4 ≤ -< 62.4	62.4 ≤ -< 125.3	≥ 125.3

Q; Quintiles

† The average daily consumption was estimated after controlling for total energy using the residual method.

Supplemental table S3. Average daily intakes (g) of the 20 food groups†

Food group (g/day)	Total		Without incident HTN		With incident HTN	
	Mean	S.D	Mean	S.D	Mean	S.D
Rice	674.4	183.1	669.9	180.2	695.0	194.8
Noodles	76.7	76.6	76.7	73.5	76.4	89.3
Bread	15.4	25.2	15.8	25.7	13.4	22.3
Sugar	14.1	15.4	14.3	15.5	13.5	14.8
Oil and fat	0.1	0.4	0.1	0.4	0.1	0.4
Potatoes	22.9	27.9	23.0	28.2	22.3	26.4
Soybean	41.4	43.6	41.6	44.0	40.8	41.8
Nuts and seeds	0.8	2.5	0.8	2.6	0.7	2.2
Kimchi	202.9	139.6	202.7	139.3	203.8	141.1
Vegetables	128.0	109.1	128.2	110.4	127.0	103.3
Mushrooms	7.9	11.5	8.0	11.6	7.5	10.9
Fruit	266.1	279.3	266.8	280.1	263.2	275.7
Meat	57.1	48.2	58.2	48.2	51.7	48.0
Eggs	12.5	15.2	12.7	15.4	11.6	14.4
Fish	27.5	28.2	27.6	28.8	27.0	25.8
Shellfish	12.4	15.4	12.5	15.4	12.0	15.5
Salted seafood	2.0	4.8	1.9	4.7	2.2	5.2
Seaweeds	1.9	1.9	1.9	1.9	1.8	1.8
Dairy products	110.5	127.3	112.0	128.2	103.4	122.8
Drinks	77.4	102.2	77.6	103.1	76.2	98.0
Total energy (kcal)	1952.8	620.0	1951.2	617.7	1960.4	630.4
Carbohydrate (%)	70.9	6.9	70.7	7.0	71.6	6.6
Protein (%)	13.5	2.4	13.5	2.4	13.4	2.3
Fat (%)	14.6	5.3	14.7	5.4	13.9	5.2

SD, standard deviation, HTN, hypertension

†The average daily consumption was estimated after controlling for total energy using the residual method.
